# Sixteen New Complete Plastid Genomes in the Tribe Loteae (Leguminosae): Structure and Phylogenetic Analysis

**DOI:** 10.3390/plants14040618

**Published:** 2025-02-18

**Authors:** Tahir H. Samigullin, Yury O. Kopylov-Guskov, Olga V. Nikitina, Anastasiya A. Krinitsina, Svetlana V. Polevova, Tatiana E. Kramina

**Affiliations:** 1 A.N. Belozersky Institute of Physico-Chemical Biology, Lomonosov Moscow State University, Leninskie Gory, 119991 Moscow, Russia; 2 Department of Higher Plants, Biological Faculty, Lomonosov Moscow State University, GSP-1, Leninskie Gory, 119234 Moscow, Russia; yurez-kg@yandex.ru (Y.O.K.-G.); chesnokovaov@my.msu.ru (O.V.N.); ankrina@gmail.com (A.A.K.); svetlanapolevova@mail.ru (S.V.P.); tkramina@msu-botany.ru (T.E.K.); 3 Faculty of Biology, Shenzhen MSU-BIT University, No. 1, International University Park Road, Dayun New Town, Longgang, Shenzhen 518172, China

**Keywords:** *Acmispon*, *Anthyllis*, *Coronilla*, *Hippocrepis*, *Lotus*, *Ornithopus*, plastid genome

## Abstract

The tribe Loteae (Papilioniodeae-Leguminosae), according to plastid data, belongs to the Robinioid clade, which also includes the tribes Robinieae and Sesbanieae. The tribe Loteae contains 16 genera and about two hundred seventy-five species, of which the plastid genomes of five species have been studied to date. The main objectives of our study were to obtain new information on the plastid genome structure of the Loteae representatives in order to assess plastid genome variability and reconstruct phylogenetic relationships within the tribe Loteae. We performed sequencing, assembly, structural and phylogenetic analyses of the Loteae plastid genomes. All assembled Loteae plastomes showed a quadripartite structure with an overall length ranging from 150,069 to 152,206 bp and showed relative stability of inverted repeat borders. The Loteae plastomes demonstrated full collinearity; the most variable sites of the studied plastomes were found in *pet*N-*trn*C and *rps*16-*acc*D spacers from the LSC region and in the *ycf*1 gene within the SSC. All inferred relationships attained maximal support with the *Hippocrepis* lineage separated first, followed by *Coronilla* and *Anthyllis*; *Lotus* is a sister group to the clade *Acmispon* + *Ornithopus*. In this study, completely resolved relationships representing a backbone of plastid phylogeny were produced. The obtained results demonstrated that plastid genomes in the tribe Loteae are structurally conservative in contrast to the closely related tribes Robinieae and Sesbanieae.

## 1. Introduction

The tribe Loteae (Papilionoideae, Fabaceae), according to recent data, includes the tribe Coronilleae [1] and comprises 16 genera and about 275 species of legumes [2]. The tribe combines annual and perennial herbs, semishrubs and rarely shrubs naturally distributed in Eurasia, Africa, Australia and North and South America, with centers of species diversity in the Mediterranean region and California [3,4].

The molecular phylogeny of the tribe Loteae was reconstructed using the nrITS marker alone [1,5] or in combination with morphology [4]. Separate phylogenetic studies have been devoted to certain genera of the tribe: *Ornithopus* L. [6], *Dorycnopsis* Boiss. [7], *Podolotus* Benth. [8], *Anthyllis* L. [9,10], *Coronilla* L. [11] and *Lotus* L. [12,13,14,15]. These studies usually included individual representatives of other genera of the tribe Loteae but did not cover the entire tribe. The phylogenetic reconstructions were based either on nrITS alone [6,7,9,11] or on nrDNA and cpDNA regions [8,10,14,15]. At the current stage, the multi-species genera of the tribe seem monophyletic, and a close affinity of certain genera composing four main evolutionary lineages (i.e., *Lotus* (incl. *Tripodion*, *Cytisopsis* and *Hammatolobium*) + *Acmispon* (incl. *Syrmatium* and *Ottleya*) + *Ornithopus + Hosakia, Anthyllis + Antopetitia*, *Coronilla + Podolotus*, *Hippocrepis + Scorpiurus*) is not questioned, but the earliest radiation events and relationships between the lineages remain ambiguous. In addition, partial incongruence of tree topologies reconstructed separately by nuclear and plastid regions was demonstrated in the phylogeny of *Anthyllis* [10] and *Lotus* [14].

Many phylogenetic studies of various plant taxa were focused on incongruent tree topologies among different genomic compartments because these genes evolve independently and are influenced by biological processes [16]. The conflicts may also occur among plastid genes or trees constructed by different plastid sequences. The possible reasons for them may include heteroplasmy and heteroplasmic recombination; the transfer of genes among plastid, mitochondrial and nuclear genomes; positive selection; and differences in GC content [16]. Additionally, the slower evolution of plastid sequences compared to nuclear ones often leads to a lack of proper resolution in the relationship between individual taxa in phylogenies reconstructed by plastid markers, which can be overcome by analyzing complete sequences of plastids.

The number of land plant species with known complete plastid genomes is approaching 50,000. Plastomes are generally conservative in organization and usually comprise four compartments: a large single-copy region (LSC, large single-copy region), a small single-copy region (SSC, small single-copy region) and two inverted repeats separating them (IRs, inverted repeats). However, in several plant lineages, the plastomes exhibit significant variability.

Despite the growth in research on sequencing the whole plastid genomes in the legume family, the degree of study remains low. Only about 24% (184 out of 770) of genera and about 1.5% (319 out of 22,000) of legume species had complete plastid genomes published in GenBank by 2021 [17]. The legume family has undergone numerous rearrangements in the plastomes, such as inversions, expansion, contraction of the genome, loss and occasional regain of a typical inverted repeat, loss of genes and introns, and accumulation of repeats [18]. Some of these changes may mark a particular group, while others occur independently in different clades [18]. The largest number of such changes was noted in the subfamily Papilionoideae. Many of the changes are related specifically to inverted repeats (IRs).

The Loteae tribe belongs to the so-called “50-kb inversion clade”, whose members have an inversion of about 50 kb in the LSC of the plastome. Within this clade, Loteae belongs to the so-called NPAAA (non-protein amino acid accumulating) clade, which includes the most economically significant cultivated legume species [19]. Molecular phylogenetic studies on the plastid site *trn*K-*mat*K clearly show that the tribe Loteae belongs to the Robinioid clade. The members of the Robinioid clade (i.e., the tribes Robinieae, Sesbanieae and Loteae) have been shown to possess both inverted repeats in their plastomes, in contrast to its sister group, the so-called IRLC (Inverted Repeat-Lacking Clade), whose members lack one of the IRs. The genus *Sesbania* Adans. distributed in the Paleo- and Neotropics appears sister to the tribe Loteae, while the tribe Robinieae, which includes several genera of woody, shrubby and sometimes herbaceous plants from the tropics and subtropics, is a sister group to “Loteae plus *Sesbania*” in many previous studies [19,20,21,22]. However, in more recent phyloplastomic analyses, Robinieae was more closely related to Loteae than Sesbanieae [17,18]. Both *Robinia* L. and *Sesbania* revealed the presence of structural rearrangements of the plastome. *Sesbania drummondii* (Rydb.) Cory, *S. cannabina* (Retz.) Poir. and *S. grandiflora* (L.) Poir. have a 50-kilobase reversion, which distinguishes them from all representatives of the “50-kb inversion clade” [18]. *Robinia pseudoacacia* L. possesses a 36 kb inversion in the LSC [23].

Until recently, plastid genomes in the Loteae tribe were known only for a small number of species: *Lotus japonicus* (Regel) K.Larsen [24], *L. corniculatus* L. [25], *Coronilla varia* L. [26], *C. valentina* L. ssp. *glauca* (L.) Batt. and *Anthyllis barba-jovis* L. [27]. It is noteworthy that these Loteae plastomes did not exhibit structural rearrangements. At the same time, for most genera of the tribe, there are no data on complete plastomes yet. This study aimed to obtain new information on the plastid genome structure of the Loteae representatives in order to assess plastid genome variability and reconstruct phylogenetic relationships within the tribe Loteae. With this purpose, we performed the following: 1. sequencing, assembly and annotation of 13 complete plastid genomes of representatives of large genera of the tribe Loteae (i.e., *Acmispon*, *Hippocrepis* and *Lotus*) and *Robinia pseudoacacia* using our own data; 2. assembly and annotation of three (*Anthyllis vulneraria*, *Hippocrepis emerus*, and *Ornithopus perpusillus*) plastomes using GenBank sequence read archive (SRA) data; 3. structural and phylogenetic analyses of the assembled plastomes.

## 2. Results

### 2.1. Comparative Analysis of the Loteae Plastomes

All sixteen newly assembled Loteae plastomes showed a quadripartite structure with an overall length ranging from 150,069 (*Lotus tetragonolobus*) to 152,206 (*Acmispon glaber*) base pairs (bp) (Table 1). The mean coverage depth of the plastomes was as follows: *Acmispon americanus* ~210×, *A. glaber* ~64×, *A. parviflorus* ~120×, *Anthyllis vulneraria* ~394×, *Hippocrepis biflora* ~87×, *H. ciliata* ~82×, *H. emerus* ~234×, *Ornithopus perpusillus* ~68×, *Lotus graecus* ~22×, *L. palustris* ~36×, Lotus dorycnium* ~485×, *Lotus herbaceus* ~33×, *Lotus hirsutus* ~39×, Lotus tetragonolobus* ~352×, Lotus conjugatus* ~1940×, Lotus ornithopodioides* ~823×, *Robinia pseudoacacia* ~660× (*—the mean coverage depth was assessed in Oxford nanopore reads). The LSC regions ranged from 81,319 bp (*Hippocrepis biflora*) to 83,583 bp (*Acmispon glaber*) in size, whereas the SSC ranged from 18,171 bp (*Acmispon parviflorus*) to 18,348 bp (*Hippocrepis emerus*); the pair of inverted repeats separated by the small single copy region ranged from 24,961 bp (*Ornithopus perpusillus*) to 25,317 bp (*Hippocrepis ciliata*).

**Figure 1:**
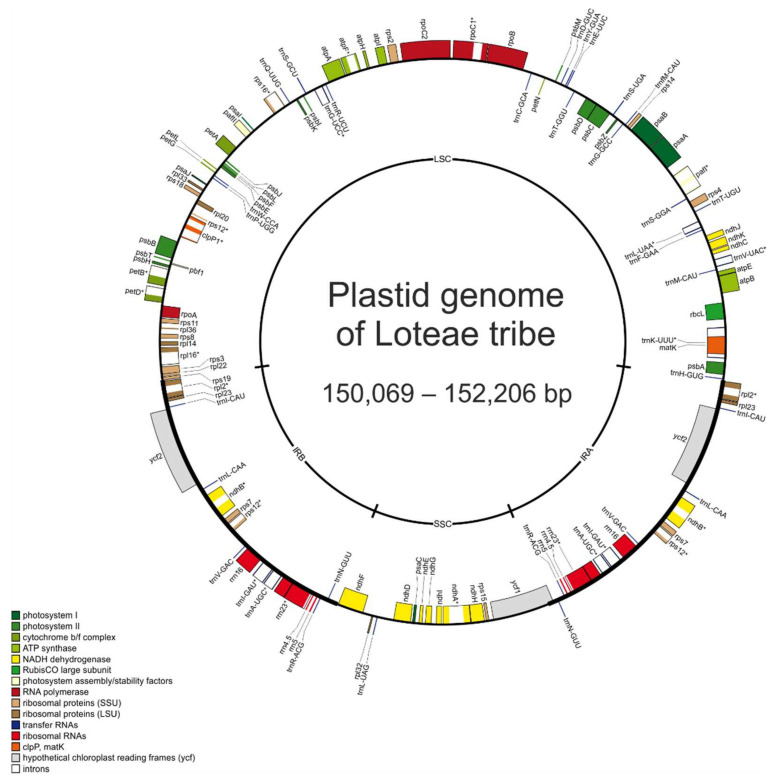
A circular genome map of the Loteae plastid genomes. Genes within the circle are transcribed clockwise, while those outside the circle are transcribed counterclockwise. Genes belonging to different functional groups are shown with various colors. Intron containing genes are marked with asterisks.

The overall GC content varied slightly between 35.8% and 36.23%. The sixteen Loteae plastomes contained 127 genes, including 82 protein-coding genes, 37 tRNA genes, and eight rRNA genes (Figure 1). The plastome of *Robinia* also possesses the quadripartite structure with all compartments being longer than those of the Loteae representatives (Table 1), and it does not contain the *rps*16 gene, like conspecific plastomes (accessions NC_026684 and MT120809).

**Figure 2:**
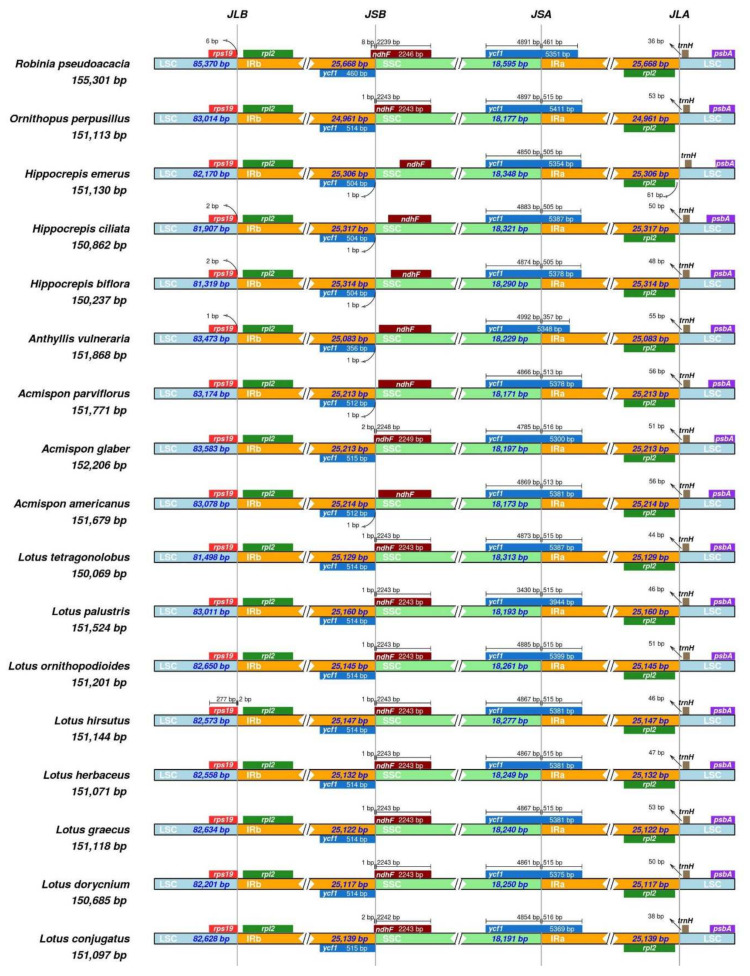
IR-SC junction positions in plastomes of 16 species of Loteae and *Robinia pseudoacacia*.

All assembled plastomes showed relative stability of inverted repeat borders with no substantial shifts (Figure 2). Thus, LSC/IRa (JLA), SSC/IRa (JSA) and LSC/IRb (JLB) junctions were found in the *rpl*2-*trn*H spacer, in the *ycf*1 gene and in the *rps*19-*rpl*2 spacer (except for *Lotus hirsutus* plastome with JLB within *rps*19), respectively. In the plastomes of *Anthyllis*, all *Hippocrepis* species and two *Acmispon* species (*A. parviflorus* and *A. americanus*), the SSC/IRb junction (JSB) was placed between the *ycf*1 fragment and *ndh*F gene, while in the plastomes of *Robinia*, *Ornithopus*, all *Lotus* species and *Acmispon glaber*, the JSB crossed the 3` end of the *ndh*F gene.

All annotated genes in Loteae plastomes retained the same relative position, suggesting full collinearity (Supplementary Figure S1). A small number of perfect dispersed repeats (with a length exceeding 30 bp) was detected, with their relative length around 1% of the total plastome (Table 2).

**Figure 3:**
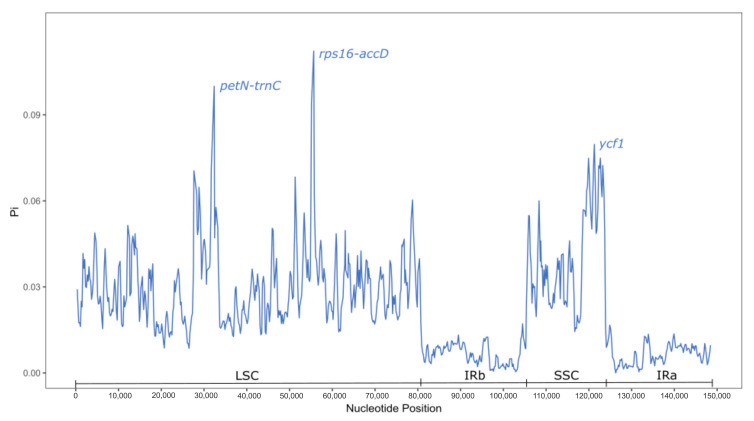
Sliding window analysis of nucleotide diversity (Pi) along the whole plastome for the 16 aligned plastomes of Loteae.

The most variable sites of the studied plastomes were found in the *pet*N*-trn*C and *rps*16*-acc*D spacers from the LSC region and in the *ycf*1 gene within the SSC (Figure 3).

### 2.2. Phylogenetic Analysis

**Figure 4:**
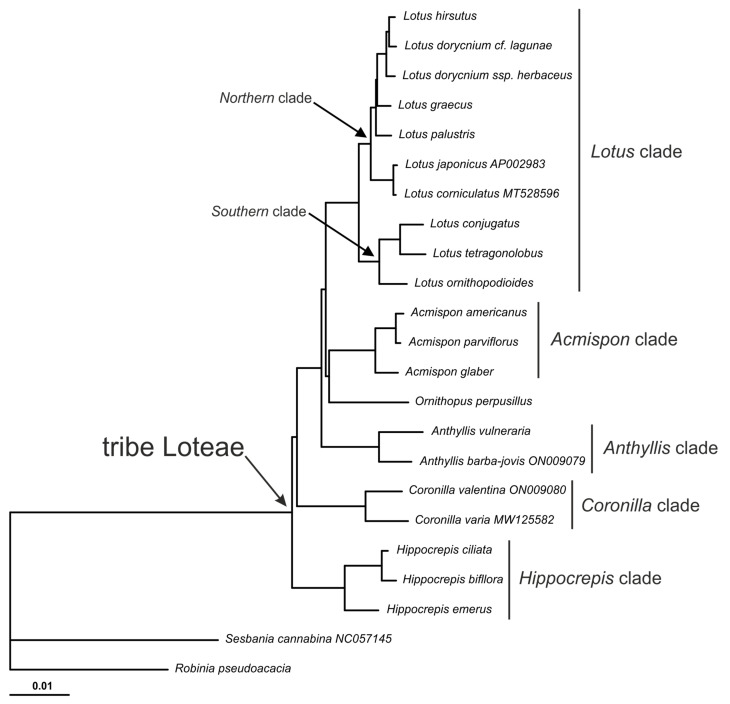
The most probable phylogenetic tree (posterior probability PP = 1.0) reconstructed in Bayesian analysis of 23 complete plastome sequences. All internal branches gained highest support (PP = 1, UFB = 100%); scale bar corresponds to 0.01 substitution per site.

Bayesian inference resulted in the only tree (with posterior probability = 1), and, in the ML analysis, the same topology was reconstructed with all the clades attaining maximal support; therefore, only the Bayesian tree is presented (Figure 4). All genera represented by two or more species have formed genus-specific clades, namely the *Hippocrepis* clade, *Coronilla* clade, *Anthyllis* clade, *Lotus* clade and *Acmispon* clade. The genus *Ornithopus*, represented in this study by a single species, *O. perpusillus*, is in a sister position to the *Acmispon* clade. The *Acmispon* + *Ornithopus* clade is, in turn, sister to the *Lotus* clade. The *Lotus* clade is subdivided into the so-called Northern clade, which includes representatives of sections *Lotus*, *Dorycnium* and *Bonjeanea*, and the Southern clade, represented in this study by representatives of sections *Lotea* and *Tetragonolobus*. The *Anthyllis* clade is sister to the ((*Acmispon* + *Ornithopus*) + *Lotus*) clade, and *Coronilla* is sister to the (((*Acmispon* + *Ornithopus*) + *Lotus*) + *Anthyllis*) clade. The *Hippocrepis* clade is sister to the clade comprising all other studied genera. Thus, among the sampled specimens, the genus *Hippocrepis* is the most distant from other genera of Loteae according to the whole plastome sequence (Figure 4).

## 3. Discussion

For this study, we have assembled 14 complete plastid genomes using our own data, and another three plastomes have been assembled from GenBank SRA, sampling species from five genera of the tribe Loteae, namely *Hippocrepis*, *Anthyllis*, *Lotus*, *Acmispon* and *Ornithopus*, and *Robinia pseudoacacia* from the tribe Robinieae. With the inclusion of *Coronilla* complete plastome data from GeneBank, the number of Loteae genera studied increased to six out of sixteen accepted genera.

As a whole, the studied plastomes of the tribe Loteae turned out to be very conservative both in their general structure and the gene composition, and they all share the same order of the genes as many unrearranged “50-kb inversion clade” plastomes.

The sampled Loteae plastome length variation was also very moderate. As is known, large length variations in the plastomes of photosynthetic angiosperms may be associated either with the loss of inverted repeats or a significant expansion/contraction of inverted repeats (e.g., in Leguminosae [18,28], Geraniaceae [29,30], Apiaceae [31,32], etc.), and none of these events have been observed in the Loteae plastomes to date.

Major structural rearrangements have also not been found, unlike the *Robinia* and *Sesbania* genera from related tribes [18,22]. The newly assembled plastome of *Robinia pseudoacacia* showed complete collinearity with two conspecific plastomes that have lost the *rps*16 gene (GenBank accessions NC_026684 and MT120809) but differs from both in length, implying possible remarkable intraspecific plastome length heterogeneity in *Robinia pseudoacacia*. It is supposed that abundance in dispersed repeats facilitates plastomic rearrangements [33,34]. However, the direct causal effect is not obvious; the presence of dispersed repeats is rather a necessary but not sufficient condition for inversion occurrence, and the similar relative length of dispersed repeats in the *Robinia* and Loteae plastomes (Table 2) supports this point of view (see also discussion in [18]).

Comparative genomic analysis revealed several regions (*pet*N-*trn*C and *rps*16-*acc*D spacers from the LSC region, and in the *ycf*1 gene within the SSC) as the most variable and potentially useful for phylogenetic purposes. It has been shown earlier that variation in the *ycf*1 gene is associated with adaptations to high-altitude conditions (*Saxifraga* species [35]) or low-light conditions (the tribe Oenantheae of the Umbelliferae family [36]). However, among the studied representatives of the tribe Loteae, there are no high-altitude species or species from shady habitats. It is highly likely that some of the variability in this gene in the Loteae plastomes is associated with other factors.

Though our study covered only a few species from each of the multi-species genera, which contain from five (*Ornithopus*) to about one hundred twenty-five (*Lotus*) currently accepted species [2], the most probable tree inferred represents the relationships of the main evolutionary lineages of the Loteae tribe. Despite incomplete sampling of Loteae taxa in this study, we found several important correlations between the tree topology based on complete plastomes and those from previous studies, which were based on a limited number of plastid DNA markers [10] or a combination of ITS and plastid markers [8]. The first important topological similarity is the sister relationships between the clade (*Lotus* + *Tripodion* + *Cytisopsis* + *Hammatolobium*) and the primarily American clade (*Acmispon* (incl. *Syrmatium* and *Ottleya*) + *Hosackia* + *Ornithopus* + *Dorycnopsis* + *Kebirita*) [10]. A similar clustering pattern was found in the study by Degtjareva et al. [8] based on a combined dataset of ITS and two plastid markers. In the present study, the first clade includes only *Lotus*, and the second includes *Acmispon* and *Ornithopus*; however, their sister relationships are highly supported. *Lotus* species have split into two highly supported clades: the Northern clade (with a presumable ancestral area in Europe and North Asia) and the Southern clade (with the corresponding area in Macaronesia, Africa and S Asia). This subdivision is consistent with the results obtained previously from a more representative set of species based on two plastid markers only [14].

The second feature worth noting is the early divergence of *Hippocrepis* within Loteae. In the study by Degtjareva et al. [10], the clade (*Hippocrepis* + *Scorpiurus*) seemed to be the earliest diverged group of Loteae, following from the Bayesian inference analysis based on a set of three plastid markers, but the low supported position of the clade (*Coronilla* + *Podolotus*) left open the question of the basalmost branch. In the present study, the first diverged clade of Loteae is clearly represented by *Hippocrepis*, not *Coronilla*. The inclusion of the remaining genera of the Loteae, including monotypic genera, in future studies of complete plastid genomes will clarify the details and the order of divergence within the main phylogenetic lineages of the tribe Loteae.

## 4. Materials and Methods

### 4.1. Taxon Sampling and Sequencing

For the analysis of the whole plastid genome, we chose the following representatives of the tribe Loteae: *Acmispon americanus* (Nutt.) Rydb., *A. glaber* (Vogel) Brouillet ssp. *glaber*, *A. parviflorus* (Benth.) D.D.Sokoloff, *Hippocrepis biflora* Spreng., *H. ciliata* Willd., *Lotus graecus* L., *L. palustris* Willd., *L. dorycnium* L. ssp. *lagunae*, *Lotus dorycnium* ssp. *herbaceus* (Vill.) Kramina & D.D. Sokoloff, *L. hirsutus* L., *L. tetragonolobus* L., *L. conjugatus* L., and *L. ornithopodioides* L. The leaves of *Acmispon americanus*, *A. glaber*, *A. parviflorus*, *Lotus palustris*, *L. graecus*, *L. dorycnium* ssp. *lagunae*, *Lotus dorycnium* ssp. *herbaceus*, *L. hirsutus*, *L. tetragonolobus*, *L. conjugatus* and *L. ornithopodioides*, which were sampled for DNA isolation from living plants grown in the greenhouse of the Lomonosov Moscow State University (see Table 3 for the origin of seed material). The leaves of *Hippocrepis ciliata* and *H. biflora* were taken from silica-dried material collected in the wild (see Table 3 for voucher information).

The total genomic DNA was extracted from fresh (ca. 100 mg of leaf tissues) or silica-dried (ca. 20 mg of leaf tissue) leaves with NucleoSpin Plant II kit (Macherey-Nagel, Düren, Germany) according to the manufacturer’s instructions. Fragmentation of DNA was performed using an S220 focused-ultrasonicator (Covaris, Woburn, MA, USA), and DNA purification was performed using Agencourt AMPure XP beads (Beckman Coulter Co., Brea, CA, USA). Paired-end Illumina libraries were constructed with the NEBNext Ultra II DNA Library Prep Kit for Illumina (New England Biolabs, Ipswich, MA, USA) according to the manufacturer’s protocol. The library was sequenced using an Illumina Nextseq500 instrument with a read length of 75 bp in paired-end mode.

The total genomic DNA from four species (*Lotus tetragonolobus, Lotus conjugatus* ssp. *requienii*, *Lotus ornithopodioides* and *Lotus dorycnium* ssp. *lagunae*) was additionally sequenced using Oxford nanopore technology. DNA Oxford nanopore libraries were prepared with Native Barcoding Kit 24 V14 (ONT, Oxford, UK) according to the manufacturer’s pcr-free protocol. The DNA library preparation consists of several steps: (1) DNA repair (FFPE) and end-prep for optimizing DNA quality; (2) preparing DNA sequence ends for barcode and adapter attachment, ligate Native barcodes supplied in the kit, and ligate sequencing adapters (3) preparing flow cell (R10.4.1) for sequencing (priming the flow cell). The DNA libraries were loaded into the R10.4.1 MinION Flowcell, and sequencing was performed using MinKnow software version 24.06.10 from ONT.

### 4.2. Chloroplast Genome Assembly and Annotation

The resulting Illumina reads were processed using Trimmomatic version 0.39 [37], and de novo assembly was performed using SPAdes toolkit version 4.0.0 [38]. Some scaffolds of the assembly of *Acmispon parviflorus*, *Hippocrepis biflora*, *H. ciliata*, *Lotus graecus*, and *L. palustris* contained stretches of undetermined bases (N), which were clarified by Sanger sequencing. Primers for amplification and sequencing are presented in Supplementary Table S1.

Oxford nanopore reads were processed with Canu assembler version 2.2 [39] and polished with Illumina reads using Polypolish tool version 0.6.0 [40].

Assembled scaffolds showing homology to plastid genomes were joined by overlapping ends.

In addition, the raw data of three representatives of the tribe Loteae *Anthyllis vulneraria* L., *Hippocrepis emerus* (L.) Lassen and *Ornithopus perpusillus* L. were retrieved from Sequence Read Archive in GenBank (accessions ERR5554795, ERR5529366 and ERR5529506, respectively), and the complete plastome sequences were assembled as described above (deposited in GenBank, accessions BK068666, BK068667 and BK068668, respectively).

To check the accuracy of assembly, trimmed paired reads were mapped to the whole assembled plastome followed by manual inspection in Tablet version 1.21.02.08 [41].

Plastome annotations were carried out with the web application GeSeq version 2.0.3 [42] and inspected using Artemis annotation tool version 16.0.0 [43]. Plastome gene maps were drawn using OGDraw version 1.3.1 [44], and IR border positions were visualized with IRscope version 0.1 tool [45]. Dispersed repeat content was explored using the repeat finder module in the Unipro UGENE package version 37.0 [46] with minimal length of perfect repeats restricted to 30 bases.

### 4.3. Comparative Analysis of the Loteae Plastomes

Plastome sequences were aligned using MAFFT version 7.471 [47] and inspected manually in Bioedit version 7.2.5 [48]. Regions where positional homology could not be firmly determined were excluded, along with the gap-rich positions and regions containing small inversions. The nucleotide divergence values (Pi) of plastid genomes were assessed in DnaSP program version 5.0 [49], with the window length and sliding step size set to 600 and 200 bp, respectively. Collinearity of the assembled plastomes of the representatives of Loteae together with two outgroups (*Robinia pseudoacacia* and *Sesbania cannabina*) was estimated using the Mauve program version 20150226 [50].

### 4.4. Phylogenetic Analysis

For phylogenetic analyses, seventeen newly assembled plastomes were combined with plastomes of *Lotus japonicus* (GenBank accession AP002983), *L. corniculatus* (MT528596), *Coronilla varia* (MW125582), *C. valentina* (ON009080), *Anthyllis barba-jovis* (ON009079) and *Sesbania cannabina* (Retz.) Poir. (NC_057145).

Phylogenetic analyses were performed for the whole genome alignment using Bayesian approach and maximum likelihood method. In all analyses, the plastomes of *Robinia* and *Sesbania* were specified as outgroups. The Bayesian phylogenetic reconstruction was performed via MrBayes v.3.2.6 [51] using four simultaneous runs of 20 million generations and four chains sampling every 1000th generation. The first million generations were discarded as burn-in, and effective sample size was evaluated using Tracer v.1.7.1 [52]. The effective sample sizes were > 200 for all statistics in all datasets, suggesting that the run length was adequate. The maximum likelihood (ML) phylogenetic reconstruction was performed by IQ-tree version 2.1.1 [53], and internal branch support was assessed with the ultrafast bootstrap approximation [54] using 10 thousand replications. To account for differences in variability and nucleotide composition across plastome compartments, LSC, SSC and inverted repeat sequences were treated as separate partitions with unlinked parameters of GTR + Г model according to the best scheme found in PartitionFinder version 2.1.1 [55].

## 5. Conclusions

The new data on 16 plastid genomes have expanded our knowledge of the characteristics of Loteae plastomes and demonstrated their structural conservation in contrast with the closely related tribes Robinieae and Sesbanieae. Phylogenetic analyses of all available Loteae plastome data resulted in highly supported and completely resolved relationships representing a backbone of plastid phylogeny.

## Figures and Tables

**Table 1:** Summary of 16 complete plastomes of Loteae and *Robinia pseudoacacia*.

Taxon	Total cpDNA Size (bp)	Length of LSC Region (bp)	Length of IR Region (bp)	Length of SSC Region (bp)	Total GC Content (%)
*Acmispon americanus*	151,679	83,078	25,214	18,173	35.80
*Acmispon parviflorus*	151,771	83,174	25,213	18,171	35.80
*Acmispon glaber* ssp. *glaber*	152,206	83,583	25,213	18,197	35.80
*Ornithopus perpusillus*	151,113	83,014	24,961	18,177	36.02
*Lotus graecus*	151,118	82,634	25,122	18,240	36.04
*Lotus palustris*	151,524	83,011	25,160	18,193	35.95
*Lotus dorycnium* ssp. *lagunae*	150,685	82,201	25,117	18,250	36.02
*Lotus dorycnium* ssp. *herbaceus*	151,071	82,558	25,132	18,249	36.01
*Lotus hirsutus*	151,144	82,573	25,147	18,277	36.00
*Lotus tetragonolobus*	150,069	81,498	25,129	18,313	36.21
*Lotus conjugatus* ssp. *requienii*	151,097	82,628	25,139	18,191	36.11
*Lotus ornithopodioides*	151,201	82,650	25,145	18,261	36.04
*Anthyllis vulneraria*	151,868	83,473	25,083	18,229	35.94
*Hippocrepis biflora*	150,237	81,319	25,314	18,290	36.23
*Hippocrepis ciliata*	150,862	81,908	25,317	18,320	36.18
*Hippocrepis emerus*	151,130	82,170	25,306	18,348	36.04
*Robinia pseudoacacia*	155,301	85,370	25,668	18,595	35.90

**Table 2:** Statistics of perfect dispersed repeats (30 bp and longer) in the plastomes of 16 species of Loteae and *Robinia pseudoacacia*.

	Dispersed Repeats Number		Direct/Inverted Repeat Length, bp	Relative Length, % of Plastome Length
	Direct	Inverted		
*Acmispon americanus*	4	1	629/39	0.88
*Acmispon glaber* ssp. *glaber*	6	2	706/77	1.02
*Acmispon parviflorus*	5	4	675/140	1.08
*Anthyllis vulneraria*	5	3	622/111	0.96
*Hippocrepis biflora*	6	2	744/79	1.08
*Hippocrepis ciliata*	5	3	698/109	1.06
*Hippocrepis emerus*	5	3	721/111	1.10
*Ornithopus perpusillus*	7	2	737/77	1.06
*Lotus conjugatus* ssp. *requienii*	5	2	652/77	0.96
*Lotus dorycnium* ssp. *lagunae*	5	2	654/77	0.96
*Lotus graecus*	5	2	654/77	0.96
*Lotus dorycnium ssp. herbaceus*	6	3	684/107	1.04
*Lotus hirsutus*	5	3	678/107	1.04
*Lotus ornithopodioides*	5	3	678/107	1.04
*Lotus palustris*	5	3	654/107	1.00
*Lotus tetragonolobus*	6	2	688/77	1.02
*Robinia pseudoacacia*	5	0	714/0	1.00

**Table 3:** Studied taxa, material origin, voucher information and GenBank accession numbers.

Taxon Name	Seed or Herbarium Collection Locality	Coordinates	Herbarium Voucher	GenBank Accession Number
*Acmispon americanus* (Nutt.) Rydb.	Cult. in the greenhouse of the MSU, Moscow. Origin: USA, El Cerrito, California, Z. Akulova-Barlow	37.904 N, 122.303 W	MW	PQ539379
*Acmispon glaber* (Vogel) Brouillet ssp. *glaber*	Cult. in the greenhouse of the MSU, Moscow. Origin: USA, Near Briones Reservoir, Contra Costa County, California, Z.Akulova-Barlow	37.918 N, 122.203 W	MW	PQ539367
*Acmispon parviflorus* (Benth.) D.D.Sokoloff	Cult. in the greenhouse of the MSU, Moscow. Origin: USA, Near Albion, Mendocino County, California, Z.Akulova-Barlow	39.205 N, 123.701 W	MW	PQ539368
*Hippocrepis biflora* Spreng.	Crimea: Sapun-gora hill, Sevastopol, Yu. Kopylov-Guskov, 2023	44.559 N, 33.544 E	MW	PQ539370
*Hippocrepis ciliata* Willd.	Crimea: southern outskirts of Balaklava town, Yu. Kopylov-Guskov, 2023	44.492 N, 33.608 E	MW	PQ539371
*Lotus graecus* L.	Cult. in the greenhouse of the MSU, Moscow. Origin: Crimea: Vinogradnoye, mount Castell, T. Kramina & S. Polevova, 2020	44.642 N, 34.384 E	MW	PQ539373
*Lotus dorycnium ssp. herbaceus* (Vill.) Kramina & D.D. Sokoloff	Cult. in the greenhouse of the MSU, Moscow. Origin: Crimea: Ayu-Dag, T. Kramina & S. Polevova, 2020	44.566 N, 34.323 E	MW	PQ539374
*L. dorycnium* L. ssp. *lagunae* (Ceresuela & Sanchis) P. P. Ferrer & Rossellу	Cult. in the greenhouse of the MSU, Moscow. Origin: Spain, Alicante, Finestrat	38.565 N, 0.212 W	MW	PQ539369
*Lotus hirsutus* L.	Cult. in the greenhouse of the MSU, Moscow. Origin: Spain, Valencia, The Botanical Garden of the University of Valencia. 2019. ES-0-VAL-476-97	–	MW	PQ539375
*Lotus palustris* Willd.	Cult. in the greenhouse of the MSU, Moscow. Origin: Mediterranean Region. Cultivated in Argentina, A.M.Arambarri	–	MW	PQ539377
*L. tetragonolobus* L.	Cult. in the greenhouse of the MSU, Moscow. Origin: Mainz Bot. Garden, 388, 143-2020	–	MW	PQ539378
*L. conjugatus* L. ssp. *requienii* (Mauri ex Sanguin.) Greuter	Cult. in the greenhouse of the MSU, Moscow. Origin: Mainz Bot. Garden, 386, 143-2020	–	MW	PQ539372
*L. ornithopodioides* L.	Cult. in the greenhouse of the MSU, Moscow. Origin: Mainz Bot. Garden, 387, 143-2020	–	MW	PQ539376
*Robinia pseudoacacia* L.	Cult. in the MSU, Moscow, Russia. Origin: Eastern N. America. Collected in 2006	–	MW	PQ636879

## Data Availability

The plastome sequence data presented in this study are deposited in GenBank (accession numbers PQ539367-PQ539379, PQ636879, BK068666-BK068668). The raw data are deposited in GenBank within BioProject number PRJNA1206441, SRA accession numbers SRX27262107-SRX27262120.

## Supplementary Materials

The following supporting information can be downloaded at https://www.mdpi.com/article/10.3390/plants14040618/s1. Figure S1: Collinearity of the 16 assembled plastomes in Loteae, *Robinia pseudoacacia* and *Sesbania cannabina*; Table S1: Primers for amplification of unassembled regions of plastomes for Sanger sequencing.
