# Supplementary material for: Sixteen New Complete Plastid Genomes in the Tribe Loteae (Leguminosae): Structure and Phylogenetic Analysis
Source: Plants (Basel). 2025 Feb 18;14(4):618. doi: 10.3390/plants14040618 (PMC11859275; doi:10.3390/plants14040618)
Supplement: Supplementary file 1 [file plants-14-00618-s001.zip › Table S1.pdf]

| Primer name | Sequence | T anneal. | Species |
|---|---|---|---|
| Hbif:24086U22 (for.) | CTATATCCGCGTTTTTTCTCGT | 60-62 | *H. biflora* |
| Hbif:24502L22 (rev.) | TGCATTAGTATTTCTAGTGGGT | 60-62 | |
| Hcil:109654U22 (for.) | GGTTCGGTTAATAAAAAAGTGG | 60 | *H. ciliata, L. graecus* |
| Hcil:110211L24 (rev.) | TTTTCTGTAAATGAAAACGAATTG | 60 | |
| Apar:24461U24 (for.) | TATAGTTTAGGGATAATTTACTCA | 60 | *A. parviflorus, L. graecus, L. palustris* |
| Apar:25050L22 (rev.) | AACTAAAACATTATAGGGAGGG | 60 | |
| Lgra:14345U23 (for.) | TTCTTTTAGTCTTAGTTTTTACC | 58 | *L. graecus* |
| Lgra:14598L24 (rev.) | TTTTATATTCTTATTACTGATTCC | 58 | |
| Lgra:54057U22 (for.) | ATTGGATTCAAAAAAGCGTAGG | 60 | *L. graecus* |
| Lgra:54273L24 (rev.) | TTATAACTTTGAATCAAAAAAGGG | 60 | |
| Lpal:123674U21 (for.) | TCTGAAATTAGAAACCCACCC | 60 | *L. palustris* |
| Lpal:124270L23 (rev.) | TTCAGATAAAGATCTTATTTCCC | 60 | |
| Lpal:125175U23 (for.) | ATTCAAACTTGATACAGATTTCC | 60 | *L. palustris* |
| Lpal:125422L24 (rev.) | ATACTTAACTAAAGAATATGATCC | 60 | |
