# Supplementary figures and images for: Sixteen New Complete Plastid Genomes in the Tribe Loteae (Leguminosae): Structure and Phylogenetic Analysis
Source: Plants (Basel). 2025 Feb 18;14(4):618. doi: 10.3390/plants14040618 (PMC11859275; doi:10.3390/plants14040618)

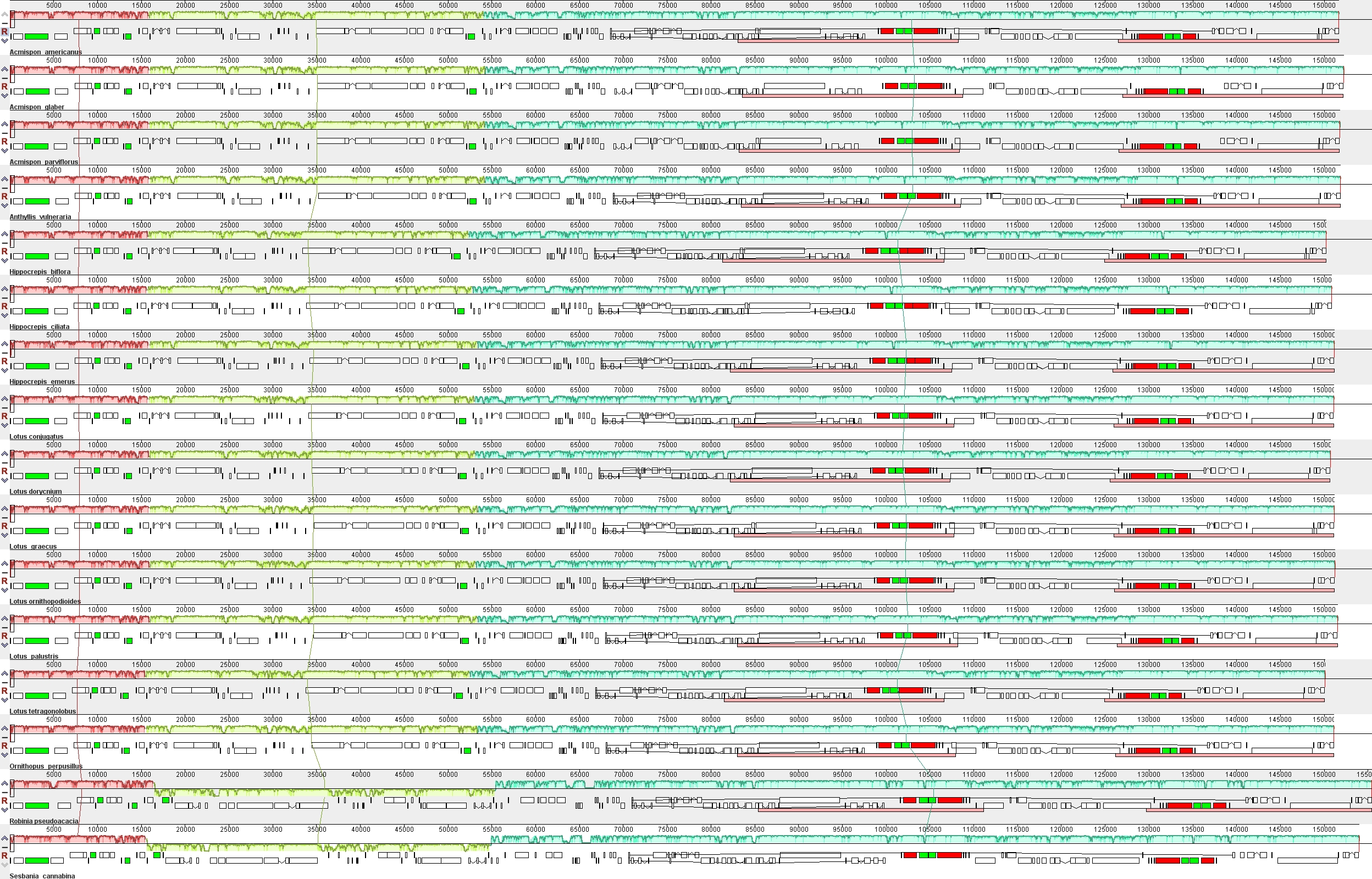

Supplement: Supplementary file 1 [file plants-14-00618-s001.zip › Figure S1.jpg]
